# Emergency department visits among rural and urban older adults: disparities in ambulatory and emergency care sensitive conditions

**DOI:** 10.1186/s12913-025-13161-2

**Published:** 2025-07-24

**Authors:** Cameron J. Gettel, Courtney Kitchen, Craig Rothenberg, Yuxiao Song, Ryan Koski-Vacirca, Katherine Schaffer, Alexander T. Janke, Nicholas M. Mohr, Margaret Greenwood-Ericksen, Arjun K. Venkatesh

**Affiliations:** 1 Department of Emergency Medicine, Yale School of Medicine, 464 Congress Ave., Suite 260, New Haven, CT 06519 USA; 2 Center for Outcomes Research and Evaluation, Yale School of Medicine, New Haven, CT USA; 3 Department of Emergency Medicine, Eastern Virginia Medical School, Norfolk, VA USA; 4 VA Center for Clinical Management Research, VA Ann Arbor Healthcare System, Ann Arbor, MI USA; 5 Department of Emergency Medicine, University of Michigan, Ann Arbor, MI USA; 6 Departments of Emergency Medicine, Anesthesia Critical Care, and Epidemiology, University of Iowa Carver College of Medicine, Iowa City, IA USA; 7 Department of Emergency Medicine, University of New Mexico School of Medicine, Albuquerque, NM USA

**Keywords:** Emergency department, Rural, Ambulatory care sensitive conditions, Emergency care sensitive conditions, Older adults

## Abstract

**Background:**

Older adults in rural geographies may be uniquely vulnerable to difficulty accessing outpatient care, and therefore more reliant on emergency department (ED) care. We compared ED utilization for ambulatory care sensitive conditions (ACSCs) and emergency care sensitive conditions (ECSCs) among rural and urban Medicare beneficiaries.

**Methods:**

We conducted a pooled cross-sectional analysis of 2016–2020 Medicare Current Beneficiary Survey data, assessing ED visitation rates for ACSCs and ECSCs. We present ED visit rates per 100 beneficiary-years and estimated logistic regression models to quantify the odds of having any ED visit, any ACSC-related ED visit, or any ECSC-related ED visit in a given year among older adults in rural and urban areas, adjusting for sociodemographic and health characteristics.

**Results:**

Our sample included 70,830 beneficiary-years, with 17,052 (24.1%) being from beneficiaries residing in rural areas. Rural beneficiaries had higher ED visit rates, with a weighted mean (SD) of 59.2 ED visits (14.1) per 100 beneficiary-years across study years, 11.5 (1.3) for ACSC-related, and 20.6 (3.5) for ECSC-related visits, compared to 43.2 (9.2), 7.2 (0.9), and 15.2 (1.9) ED visits, respectively, for urban beneficiary-years. In adjusted models, rural beneficiaries had a 49% higher odds of having an ED visit (OR: 1.49, 95% CI: 1.40–1.59), a 30% higher odds of an ACSC-related ED visit (OR: 1.30, 95% CI: 1.04–1.64), and a 26% higher odds of an ECSC-related ED visit (OR: 1.26, 95% CI: 1.05–1.50) within a given year when compared to urban counterparts.

**Conclusions:**

Rural Medicare beneficiaries consistently showed higher ED utilization for ACSCs and ECSCs compared to urban beneficiaries, highlighting potential disparities in healthcare access and a need for targeted or policy-based interventions.

**Supplementary Information:**

The online version contains supplementary material available at 10.1186/s12913-025-13161-2.

## Background

Increasing emergency department (ED) visitation among older adults reflects broader challenges in the healthcare system’s ability to meet the needs of an aging population [1–5]. Older adults are more susceptible to a range of acute and chronic conditions, making timely access to care critical for preventing complications and hospitalizations [6]. In rural areas, where healthcare infrastructure is often sparse, EDs frequently serve as the primary or only point of access for many individuals [7–9]. This reliance on emergency services may strain limited ED resources and highlights underlying issues in outpatient care availability and preventive health services [10–13]. The consequences of inadequate access to primary care are particularly severe for conditions classified as ambulatory care sensitive conditions (ACSCs), such as diabetes, hypertension, and chronic obstructive pulmonary disease. Managing these conditions effectively in outpatient settings can significantly reduce the need for related ED visits and hospitalizations [14–18].

Despite substantial research on healthcare disparities between rural and urban populations, [19–21] significant gaps remain in understanding the specific patterns of ED use among older adults, particularly for ACSCs and emergency care sensitive conditions (ECSCs) such as sepsis, heart failure, and respiratory failure [22–24]. Measuring ED utilization for ECSCs is critical because, unlike ACSCs, ECSCs reflect acute conditions for which timely emergency care is essential to prevent adverse outcomes. This distinction allows for a more nuanced understanding of the underlying drivers of rural ED use and highlights potential policy and intervention strategies. High prevalence of ECSCs in rural communities indicate a critical need to maintain access to emergency care, which is increasingly threatened by rural hospital closures [25–27]. While ACSC-related data can inform efforts to improve chronic disease management and overall health status, understanding ECSC-related ED visits is important for addressing disparities in health outcomes, addressing exacerbations of chronic diseases, and costs associated with acute hospitalizations. These efforts are especially relevant given the new rural hospital payment model, the Rural Emergency Hospital (REH), which may provide an alternative to closure while maintaining access to emergency care services.

To date, existing studies have established that rural residents often face greater barriers to accessing primary care, including fewer healthcare providers, longer travel distances, and higher rates of poverty [28–30]. These barriers contribute to higher rates of preventable hospitalizations and ED visits [31–33]. However, the literature has primarily focused on general ED utilization trends or has been limited to specific conditions or populations [34–37]. There is a lack of comprehensive analysis measuring ACSC- and ECSC-related ED visits between rural and urban older adults, thereby leaving a gap in the evidence base in understanding the role of the ED in providing care for ambulatory and emergency conditions that will inform interpretation of the effect of REH designation.

The objective of this study is to examine differences in ED utilization for ACSCs and ECSCs between rural and urban Medicare beneficiaries. Utilizing data from the Medicare Current Beneficiary Survey (MCBS) from 2016 to 2020, this study provides a robust analysis of how geographic location impacts ED use among older adults. By exploring these patterns, we aim to identify key areas where targeted interventions can improve ambulatory and hospital care access and outcomes for rural populations, ultimately enhancing the overall value of healthcare delivery for older adults in both rural and urban settings.

## Methods

### Study design and data source

We conducted a pooled cross-sectional analysis utilizing data from the MCBS from 2016 to 2020. The MCBS consists of approximately 15,000 Medicare enrollees annually, intentionally enriched for Hispanic beneficiaries and individuals over the age of 85 years. The MCBS is a continuous in-person survey with a rotating panel design of a representative national sample of both traditional Fee-for-Service (FFS) Medicare and Medicare Advantage (MA) beneficiaries. The MCBS collects self-reported health care utilization in the Survey Files, including ED visits, hospitalizations, skilled nursing care facility stays, and inpatient rehabilitation. MCBS has been used in several prior ED-based studies [38–41]. For this analysis, the unit of analysis was the beneficiary when describing the rate of ED visits for an individual year and was beneficiary-year when describing the rate of ED visits for a pooled sample across several years as a way to standardize comparisons. Since MCBS allowed follow-up of beneficiaries across several years, this approach accounted for changes in community access to care that may alter ED visitations year-to-year as well as changes in beneficiary geographic residential status over time.

The sample included all Medicare beneficiaries enrolled in the survey between January 1^st^ 2016 and December 31^st^ 2020, selected to allow for a robust examination of the effects of geographic location on ED utilization. Beneficiaries without a geographic location of residence – urban or rural – were excluded.

The Yale Institutional Review Board determined that this study was exempt secondary research for which patient informed consent was not required. Research has been conducted in accordance with the Declaration of Helsinki. Our use of MCBS data conforms to the Strengthening the Reporting of Observational Studies in Epidemiology (STROBE) reporting guidelines [42].

### Outcomes

Our primary outcome of interest was ED visit rates among Medicare beneficiaries in rural and urban areas. As a secondary outcome, we assessed the frequency of ACSC-related ED visits and ECSC-related ED visits.

We first extracted all ED visits identifiable within the MCBS claims data. We relied on a stepwise approach consistent with prior literature to identify all possible healthcare service use that could represent an ED visit [43]. We used the Outpatient Hospital Events segment from the MCBS Cost Supplement File to identify emergency care based on the Original Reported Event Type. We used outpatient facility claims and physician service, or “carrier”, claims to identify additional items reflecting emergency care respectively based on revenue center codes as well as Healthcare Common Procedure Coding System codes and “place of service” designations, consistent with prior literature detailing emergency care identification using claims data [43]. ACSC-related ED visits represent those for acute, chronic, and often considered preventable conditions, while ECSC-related ED visits represent those for conditions considered high risk for mortality if not identified and treated rapidly [17, 23]. ACSCs and ECSCs were identified using ICD-10 diagnostic codes, consistent with prior literature [18, 23].

### Covariates

The key predictor variable of interest was geographic location of residence of the Medicare beneficiary – urban or rural – as identified in the MCBS Survey files. Rurality was defined using the primary Rural Urban Commuting Area (RUCA) codes, based on the beneficiary’s address. We categorized beneficiaries residing in metropolitan areas (RUCA codes 1 to 3) as urban and beneficiaries in micropolitan and nonmetropolitan areas (RUCA codes 4 to 10) as rural. We accounted for the possibility of beneficiaries moving from an urban location of residence to a rural location of residence (or vice versa) by conducting analyses at the beneficiary-year level. We included beneficiary characteristics as covariates in the analyses which are conceptually or shown by existing to literature to be associated with ED visits. Based on the first year of available data, these included sociodemographic factors such as age, gender, marital status, and income level. Marital status Household income was categorized as high (>$40,000/year), average ($20,000-$40,000) and low (<$20,000/year), as performed previously [40]. Health-related variables included the number of chronic medical conditions (≥2 or < 2) and presence of dementia.

### Statistical analysis

We calculated descriptive statistics of the sample and examined the geographical distribution of ED visits. Separately, we estimated three logistic regression models, for whether a Medicare beneficiary had: (1) at least one ED visit, (2) at least one ACSC-related ED visit, and (3) at least one ECSC-related ED visit, controlling for beneficiary characteristics. These binary outcomes were defined as the occurrence of at least one ED visit of the specified type per beneficiary-year. Variable selection for these models was informed by a literature review to ensure alignment with established determinants of ED utilization. To assess potential multicollinearity among the predictor variables, we calculated variance inflation factors (VIFs) and found no evidence of high collinearity, with all VIFs below the standard threshold of 5. Outliers were assessed and determined to have minimal impact on the results. Analyses were conducted at the beneficiary level using R-Studio.

### Sensitivity analysis

To assess the robustness of our findings, we conducted a sensitivity analysis limited to patients who had data available across all four years of the study. This approach was designed to ensure that our primary results were not driven by patients with incomplete follow-up data. For this analysis, we utilized logistic regression models, focusing on the same outcomes examined in the primary analysis: (1) at least one ED visit, (2) at least one ACSC-related ED visit, and (3) at least one ECSC-related ED visit. The primary covariate of interest remained rural versus urban status.

## Results

From 2016-2020, our sample included 36,621 unique Medicare beneficiaries contributing a total 70,830 beneficiary-years to the analysis. The majority of beneficiaries were White (83.0%) and female (55.7%), with a median (IQR) age of 76 (68–83) years. Among the 70,830 beneficiary years, 17,052 (24.1%) were from rural areas (Table 1).

**Table 1:** Beneficiary-year level rural and urban population characteristics

**Characteristic**	Overall (*n* = 70,830)	Rural (*n* = 17,052)	Urban (*n* = 53,778
Age 75+ years, n (%)	39,970	9,203	30,767
	(56.4%)	(54.0%)	(57.2%)
Female sex, n (%)	39,806	9,439	30,367
	(56.2%)	(55.4%)	(56.5%)
Race, n (%)			
White	59,150	15,179	43,971
	(83.5%)	(89.0%)	(81.8%)
Black	7,048	1,340	5,708
	(10.0%)	(7.9%)	(10.6%)
Hispanic	2,385	114	2,271
	(3.4%)	(0.7%)	(4.2%)
Other	2,247	419	1,828
	(3.2%)	(2.5%)	(3.4%)
Income, n (%)			
Low (<$20,000)	22,507	6,151	16,356
	(31.8%)	(36.1%)	(30.4%)
Average ($20,000-$40,000)	18,919	5,102	13,817
	(26.7%)	(29.9%)	(25.7%)
High (>$40,000)	29,404	5,799	23,605
	(41.5%)	(34.0%)	(43.9%)
Married Status, n (%)	32,909	8,107	24,802
	(46.5%)	(47.5%)	(46.1%)
Dementia, n (%)	1,400	335	1,065
	(2.0%)	(2.0%)	(2.0%)
2+ Chronic Conditions^*^, n (%)	22,241	5,983	16,258
	(31.4%)	(35.1%)	(30.2%)

*Chronic conditions considered: Hypertension, Heart attack, Congestive heart failure, Stroke/brain hemorrhage, High cholesterol, Cancer (lung, colon, breast, uterus, prostate, bladder, ovary, stomach, cervix, brain, kidney, throat, blood, bone, esophagus, gall bladder, larynx, leukemia, liver, lymph nodes, mouth/tongue/lip, pancreas, rectum, soft tissue/fat, testis, thyroid), Rheumatoid arthritis, Depression, Parkinson’s disease, Emphysema/asthma/COPD, Diabetes/high blood sugar

Overall, there were 32,664 ED visits among included MCBS beneficiaries from 2016 to 2020, with rural and urban beneficiaries respectively accounting for 9,913 (30.3%) and 22,751 (69.7%) of the total ED visits. In rural areas, beneficiaries contributed 3,410 (34.4%) ECSC-related ED visits and 1,915 (19.3%) ACSC-related ED visits, with the remainder being for non-ECSCs and non-ACSCs. In urban areas, beneficiaries contributed 8,037 (35.3%) ED visits for ECSCs and 3,704 (16.3%) for ACSCs, with the remainder being for non-ECSCs and non-ACSCs.

**Fig. 1:**
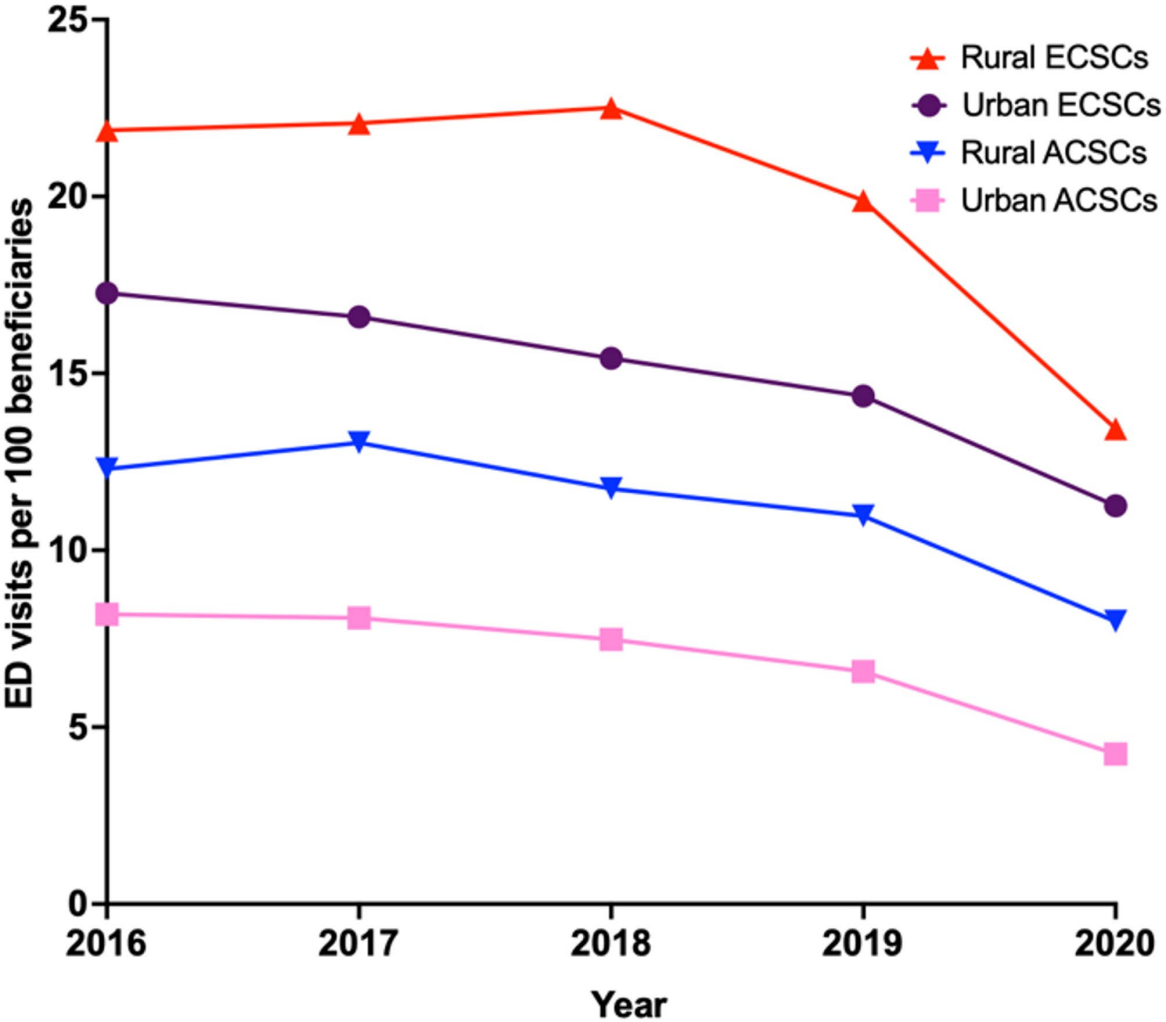
ED visit rates for rural and urban Medicare beneficiaries, 2016 to 2020. Abbreviations: ACSC – ambulatory care sensitive condition; ECSC – emergency care sensitive condition

Across the five study years, the weighted mean (SD) ED visits per 100 beneficiaries across different geographies and conditions showed distinct patterns (Fig. 1). For all ED visits, the overall weighted mean (SD) across all years was 47.1 ED visits (10.2) per 100 beneficiaries. When stratified by geography, rural areas experienced a higher weighted mean (SD) of ED visits, with 59.2 visits (14.1) per 100 beneficiaries, compared to 43.2 (9.2) in urban areas. For ACSCs, the weighted mean (SD) in rural areas was 11.5 ED visits (1.3) per 100 beneficiaries, significantly higher than the 7.2 visits (0.9) per 100 beneficiaries in urban areas. For ECSCs, rural areas had a higher weighted mean (SD) of 20.6 ED visits (3.5) per 100 beneficiaries, compared to 15.2 (1.9) in urban areas.

Assessing individual study years, there was a decline in the rate of ED visits per 100 beneficiaries across both rural and urban settings, with a more pronounced drop observed in 2020, likely reflecting the impact of the COVID-19 pandemic. In 2016, rural beneficiaries had a higher ED visit rate (63.9 per 100) compared to urban beneficiaries (49.3 per 100), and while both groups saw reductions in ED use over time, the rural population consistently exhibited higher ED visit rates. By 2020, the ED visit rate dropped to 43.7 per 100 rural beneficiaries and 31.0 per 100 urban beneficiaries, indicating a continued disparity in ED utilization between the two populations.

When focusing on ACSC-related ED visits, rural beneficiaries consistently had higher ED visit rates compared to their urban counterparts. For instance, in 2016, rural patients had 12.3 ACSC-related ED visits per 100 beneficiaries, while urban patients had 8.2 ED ACSC-related visits per 100 beneficiaries. Although there was a decline in both rural and urban ED visits related to ACSCs over the study period, rural areas saw a sharper reduction in 2020, falling to 8.0 ACSC-related ED visits per 100 beneficiaries compared to 4.2 in urban areas. Similarly, for emergency care related to ECSCs, rural beneficiaries showed consistently higher ED visit rates. In 2016, rural beneficiaries had 21.9 ECSC-related ED visits per 100 beneficiaries compared to 17.3 in urban areas. The gap between rural and urban populations persisted throughout the period, although both groups experienced reductions, with the rural rate dropping to 13.4 and the urban rate to 11.3 ECSC-related ED visits per 100 beneficiaries by 2020 (Fig. 1).

**Fig. 2:**
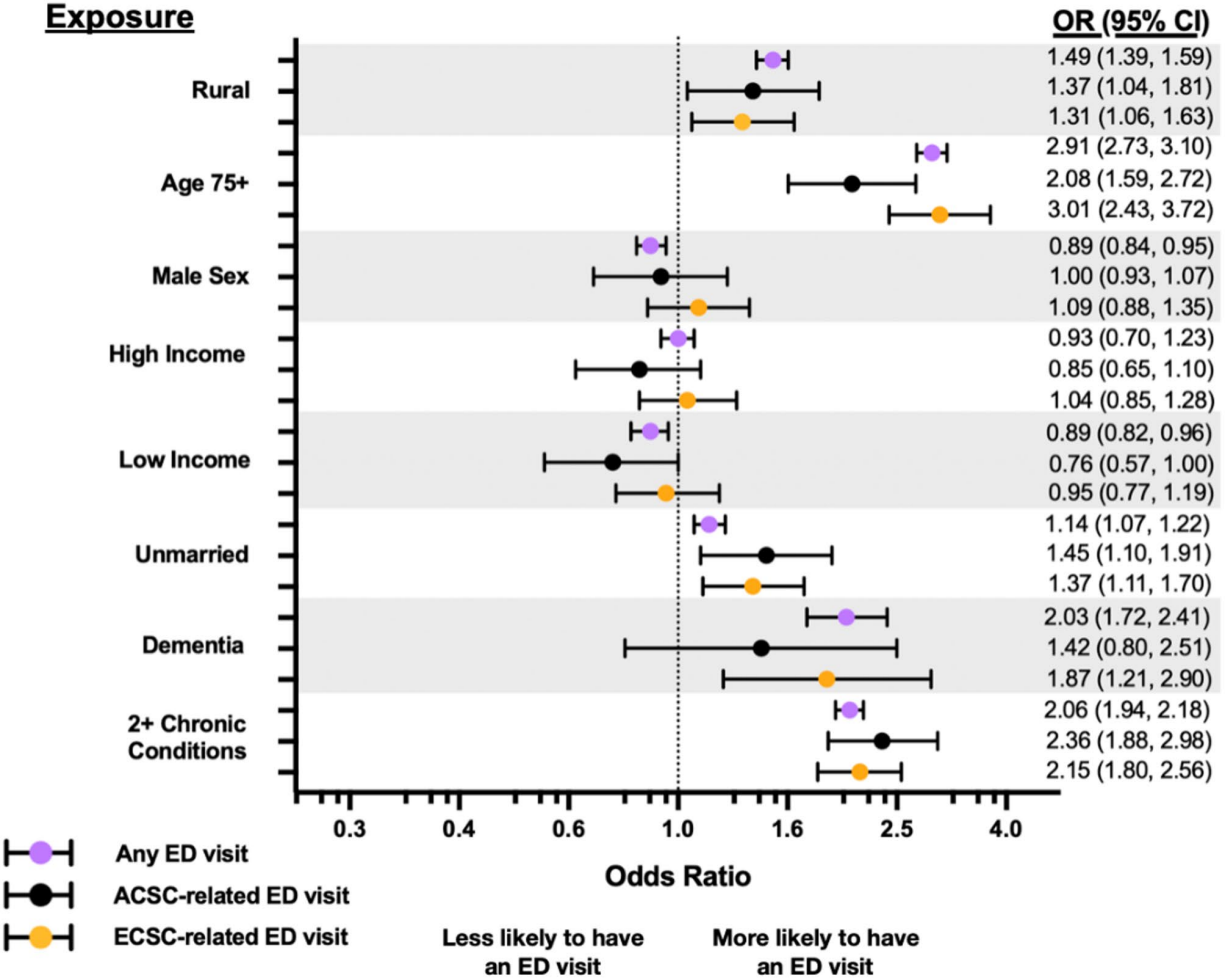
Forest plot associations of predictor variables with any ED visits, any ACSC-related ED visits, and any ECSC-related ED visits within a given beneficiary-year. *Note: An example interpretation suggests that beneficiaries residing in rural areas had an OR (CI) of 1.49 (1.39, 1.59) in meeting the outcome of having at least one ED visit within a given year, suggesting they were 49% more likely to have at least one ED visit than those residing in urban areas

In logistic regression models estimating the odds of experiencing at least one ED visit in a given year, rural residence remained associated with higher odds of ED utilization for overall visits (OR: 1.49, 95% CI: 1.39–1.59), any ACSC-related visit (OR: 1.37, 95% CI: 1.04–1.81), and any ECSC-related visit (OR: 1.31, 95% CI: 1.06–1.63). Notably, older age (75+), the presence of dementia, and having at least two other chronic conditions were strong predictors of any ED use across all models (Fig. 2, Supplemental Table 1).

The sensitivity analysis, restricted to patients with data across all four years, yielded results consistent with the primary findings. In the fully adjusted models, rural beneficiaries continued to show higher odds of any ED utilization, particularly for any ECSC-related visits (OR: 1.45, 95% CI: 1.15–1.83). Age 75 and older and having at least two chronic conditions remained significant predictors of any ED use across all models. The sensitivity analysis confirmed the robustness of the primary analysis, demonstrating consistent patterns of elevated ED utilization among rural patients across multiple outcomes, even when restricting the cohort to those with complete data over four years.

## Discussion

Our study highlights significant disparities in ED utilization patterns among older adults, particularly between rural and urban populations. Rural older adults were identified to use ED services at a notably higher rate and have a higher odds of ED use annually than their urban counterparts, even after controlling for key demographic and health-related factors. These findings suggest that geographic location plays a critical role in shaping acute care service utilization, with rural older adults potentially facing distinct challenges in accessing timely and adequate care, which may lead to greater reliance on ED services.

These results are consistent with existing literature that shows rural populations experience higher rates of ED utilization compared to urban populations [8, 44, 45]. Contributing factors such as fewer healthcare facilities, longer travel distances to primary and specialty care, and higher rates of chronic health conditions have been well-documented [28–30, 46–48]. Our study builds on this body of work by focusing specifically on older adults, a group with higher healthcare needs and greater vulnerability to access barriers. The persistence of these disparities, despite overall declining ED use among older adults, underscores the chronic nature of healthcare access issues in rural areas.

A key finding of our analysis was the higher ED utilization for ACSCs and ECSCs among rural older adults. The higher rates of ACSC-related ED visits suggest that rural older adults may struggle to access primary care or specialty ambulatory care services, leading to preventable ED visits for conditions that could be managed in outpatient settings. This is particularly concerning, as failure to manage ACSCs can result in worse health outcomes and increased healthcare costs. Similarly, the elevated rate of ECSC-related visits potentially indicates barriers to timely emergency care, which may be exacerbated by rural hospital closures and longer travel distances. It is possible that these ECSC-related visits reflect not only higher acute care needs in rural populations but also patients ‘bypassing’ rural EDs due to perceived or actual limitations in local care capacity – potentially leading to delayed or more severe presentations at larger urban centers, even in the absence of hospital closures [49]. Concordant current evidence suggests that these disproportionate ECSC-related visits may be associated with disproportionate mortality for rural patients [50]. These findings highlight the critical need for concurrently improving access to both outpatient and emergency care in rural areas to reduce preventable ED visits and improve outcomes for older adults.

The clinical and policy implications of our findings are substantial. From a clinical perspective, healthcare providers and systems serving rural populations must recognize the increased reliance on EDs among older adults and work to ensure proper follow-up care after ED visits. The recent trend of rural hospital and outpatient medical office closures has likely worsened these access issues, further limiting care options and increasing reliance on ED services for both urgent and routine care. Rural hospital closures, in particular, reduce the availability of inpatient and emergency services, forcing many older adults to travel greater distances for care [51–53]. Rural Emergency Hospitals (REHs) have been introduced as a potential solution to mitigate these access challenges by maintaining emergency services in communities where full-service hospitals may not be sustainable [54, 55]. Furthermore, policymakers and healthcare leaders should focus on expanding rural telemedicine services, improving care coordination for chronic conditions, and incentivizing clinical practice in the rural workforce.

Our study has several limitations. First, while we observed an overall decline in ED visits for ACSCs and ECSCs among older adults, our analysis did not specifically account for the broader trend of overall ED utilization over time. It is important to recognize that this reduction may be due to several factors, including changes in healthcare delivery models, improved access to preventive and primary care, and the increasing use of telehealth services – particularly in 2020, this trend was amplified given the COVID-19 pandemic which reduced national ED visits significantly. Nevertheless, despite this trend, the disparity between rural and urban populations in ED use remains pronounced, suggesting that geographic location continues to be a critical determinant of healthcare access for older adults. Second, identification of ACSC-related ED visits based on diagnosis codes has important limitations, as it may not accurately reflect whether an encounter was truly avoidable, nor does it capture the structural barriers – such as limited access to timely outpatient care – that may appropriately drive ED utilization. Finally, we attempted to account for a beneficiary changing residential geographic designation within the study timeframe, with presentation at the beneficiary-year level and being supportive of a strength of the analysis. However, we were unable to account for changes of residential geographic designations mid-year; yet we do not anticipate a systematic bias of urban to rural migration (or vice versa) mid-year that would substantively impact our findings.

## Conclusions

In conclusion, our study highlights the persistent disparities in ED utilization between rural and urban older adults. These findings suggest that rural older adults continue to face significant barriers in accessing healthcare, leading to a heavier reliance on ED services for both primary care-treatable and emergency conditions. Addressing these disparities requires concerted efforts from healthcare providers, policymakers, and community leaders to improve healthcare access and delivery in rural areas. By investing in acute care infrastructure, expanding telemedicine, and enhancing care coordination, we can ensure access to ambulatory and emergency care, particularly for older adults in rural settings.

## Supplementary Information

Supplementary Material 1.

## Data Availability

The data that support the findings of this study are available from the Centers for Medicare & Medicaid Services (CMS) but restrictions apply to the availability of these data, which were used under license for the current study, and so are not publicly available. Data are however available from the authors upon reasonable request and with permission of CMS.
