# Supplementary material for: Emergency department visits among rural and urban older adults: disparities in ambulatory and emergency care sensitive conditions
Source: BMC Health Serv Res. 2025 Jul 24;25:975. doi: 10.1186/s12913-025-13161-2 (PMC12291402; doi:10.1186/s12913-025-13161-2)
Supplement: Supplementary file 1 — Supplementary Material 1. [file 12913_2025_13161_MOESM1_ESM.pdf]

Predictor	Logistic Model		
	Estimate	95% CI	*P* value
**Outcome – at least one ED visit**			
Geography (Rural)	1.49	(1.39, 1.59)	<.001
Age (75+)	2.91	(2.73, 3.10)	<.001
Sex (Male)	0.89	(0.84, 0.95)	<.001
Income			
High	1.00	(0.93, 1.07)	0.90
Low	0.89	(0.82, 0.96)	0.002
Average	ref	-	-
Marital Status (Unmarried)	1.14	(1.07, 1.22)	<.001
Dementia	2.03	(1.72, 2.41)	<.001
2+ Chronic Conditions	2.06	(1.94, 2.18)	<.001

Predictor	Logistic Model		
	Estimate	95% CI	*P* value
**Outcome – at least one ECSC-related ED visit**			
Geography (Rural)	1.31	(1.06, 1.63)	0.01
Age (75+)	3.01	(2.43, 3.72)	<.001
Sex (Male)	1.09	(0.88, 1.35)	0.42
Income			
High	1.04	(0.85, 1.28)	0.69
Low	0.95	(0.77, 1.19)	0.68
Average	ref	-	-
Marital Status (Unmarried)	1.37	(1.11, 1.70)	0.003
Dementia	1.87	(1.21, 2.90)	0.005
2+ Chronic Conditions	2.15	(1.80, 2.56)	<.001

Predictor	Logistic Model		
	Estimate	95% CI	*P* value
**Outcome – at least one ACSC-related ED visit**			
Geography (Rural)	1.37	(1.04, 1.81)	0.03
Age (75+)	2.08	(1.59, 2.72)	<.001

Sex (Male)	0.93	(0.70, 1.23	0.61
Income			
High	0.85	(0.65, 1.10)	0.22
Low	0.76	(0.57, 1.00)	0.05
Average	ref	-	-
Marital Status (Unmarried)	1.45	(1.10, 1.91)	0.01
Dementia	1.42	(0.80, 2.51)	0.23
2+ Chronic Conditions	2.36	(1.88, 2.98)	<.001

Supplemental Table 1. Associations of predictor variables with ED visits, ECSC-related ED visits, and ACSC-related ED visits.
